# *Erratum*: Vol. 66, No. SS-25

**DOI:** 10.15585/mmwr.mm6948a10

**Published:** 2020-12-04

In the Surveillance Summary “Abortion Surveillance — United States, 2014,” data on the number of known previous induced abortions for women having abortions in this reporting year were erroneously included for New York City. These estimates did not meet reporting standards and should have been excluded from this report. When corrected, among women with abortions in the reporting year, the proportion with no previous induced abortions increased, and the proportion with one or more previous induced abortions decreased.

On page 2, the second and third sentences of the second paragraph should have read “Women with one or more previous induced abortions accounted for **42.5%** of abortions, and women with no previous abortion accounted for **57.5%**. Women with three or more previous births accounted for 13.8% of abortions, and women with three or more previous abortions accounted for **7.0%** of abortions.”

On page 10, the first paragraph should have read “Data from the **39** areas that reported the number of previous abortions for women who obtained abortions in 2014 indicate that the majority (**57.5%**) had no previous abortions, **35.5%** had one to two previous abortions, and **7.0%** had three or more previous abortions (Table 17). Among the **32** reporting areas^§§§§^ that provided data for the relevant years of comparison (2005 to 2014, 2005 versus 2009, 2010 versus 2014, and 2013 versus 2014), the percentage of women who had zero or one to two previous abortions did not change substantially over time, but the percentage of women who had three or more previous abortions increased from 2005 to 2014. Among the areas included in this comparison, **57.9%**, **35.6%**, and **6.4%** of women had zero, one to two, or three or more previous abortions, respectively, in 2005; **58.3%**, **35.1%**, and **6.6%** of women had zero, one to two, or three or more previous abortions, respectively, in 2014.” New York City should have been included in the ^§§§§^ footnote, which lists reporting areas that were not included in these estimates.

In Table 17, the line for New York City should be deleted. For the total line, the numbers and percentages should have read **224,737 (57.5), 97,093 (24.8), 41,871 (10.7), 27,256 (7.0), 390,957 (98.5)**. The * footnote should have read “Data from **39** reporting areas; excludes **13** areas (California, Connecticut, District of Columbia, Florida, Illinois, Maryland, New Hampshire, New Mexico, **New York City**, New York State, North Carolina, Wisconsin, and Wyoming) that did not report, did not report by the number of previous induced abortions, or did not meet reporting standards.” The total in the ** footnote should have been **397,042**.

